# Band 3 Erythrocyte Membrane Protein Acts as Redox Stress Sensor Leading to Its Phosphorylation by p^**72**^ Syk

**DOI:** 10.1155/2016/6051093

**Published:** 2015-12-29

**Authors:** Antonella Pantaleo, Emanuela Ferru, Maria Carmina Pau, Amina Khadjavi, Giorgia Mandili, Alessandro Mattè, Alessandra Spano, Lucia De Franceschi, Proto Pippia, Francesco Turrini

**Affiliations:** ^1^Department of Biomedical Sciences, University of Sassari, 07100 Sassari, Italy; ^2^Department of Oncology, University of Turin, 10126 Turin, Italy; ^3^Department of Medicine, Section of Internal Medicine, University of Verona, 37134 Verona, Italy; ^4^Department of Molecular Biotechnology and Life Sciences, University of Torino, 10126 Turin, Italy

## Abstract

In erythrocytes, the regulation of the redox sensitive Tyr phosphorylation of band 3 and its functions are still partially defined. A role of band 3 oxidation in regulating its own phosphorylation has been previously suggested. The current study provides evidences to support this hypothesis: (i) in intact erythrocytes, at 2 mM concentration of GSH, band 3 oxidation, and phosphorylation, Syk translocation to the membrane and Syk phosphorylation responded to the same micromolar concentrations of oxidants showing identical temporal variations; (ii) the Cys residues located in the band 3 cytoplasmic domain are 20-fold more reactive than GSH; (iii) disulfide linked band 3 cytoplasmic domain docks Syk kinase; (iv) protein Tyr phosphatases are poorly inhibited at oxidant concentrations leading to massive band 3 oxidation and phosphorylation. We also observed that hemichromes binding to band 3 determined its irreversible oxidation and phosphorylation, progressive hemolysis, and serine hyperphosphorylation of different cytoskeleton proteins. Syk inhibitor suppressed the phosphorylation of band 3 also preventing serine phosphorylation changes and hemolysis. Our data suggest that band 3 acts as redox sensor regulating its own phosphorylation and that hemichromes leading to the protracted phosphorylation of band 3 may trigger a cascade of events finally leading to hemolysis.

## 1. Introduction

Due to their function in carrying oxygen and their high iron content, red blood cells (RBCs) are constantly exposed to oxidative stress [1]. In addition, RBCs may transiently experience oxidative stress when they are exposed to ROS crossing inflammatory tissues or interacting with oxidant contained in drugs or foods [2–4]. Moreover, a number of hemolytic disorders are also known to damage the RBC membrane increasing the production of free radicals originating from denatured hemoglobin species (hemichromes), invariably present in thalassemia, sickle cell disease [5–7] or with decreased ability of RBCs to deal with extracellular oxidants as in G6PD deficiency [8].

It is noteworthy that approximately 7% of world population is affected by those mutations which have been selected by malaria.

It is well known that RBCs respond to oxidative stress with a metabolic response finalized to maximize the production of NADPH and to regenerate the stores of GSH and thioredoxin. In parallel, RBCs also respond by activating tyrosine kinases determining the tyrosine (Tyr) phosphorylation of band 3, the most abundant RBC membrane protein and the major linkage between the cytoskeleton and the lipid bilayer [9–12]. In RBCs, hyperphosphorylation of band 3 has been constantly reported in all the prooxidant hemolytic disorders [13–15] and in malaria [16, 17], but the mechanisms leading to its phosphorylation and its pathophysiological significance have been partially defined. We recently described that band 3 phosphorylation appears to be increased in intermediate thalassemia [18] and that this phenomenon is closely related to the formation of hemichromes. Band 3 phosphorylation and hemichromes formation have been also described in malaria infected RBC [19]. In both pathological situations, band 3 phosphorylation appears to play a permissive role in the release of membrane microparticles. Current knowledge appears to be still insufficient to explain the molecular details of the underlying mechanism, although some recent findings clearly indicate a role of band 3 phosphorylation in the regulation of metabolism mediated by the binding of deoxygenated hemoglobin (Hb) [20–22] and in the modification of the affinity between band 3 and ankyrin following oxidative stress [23].

The redox regulation of band 3 Tyr phosphorylation apparently involves different components. In a previous report, it has been demonstrated that oxidized band 3 is selectively phosphorylated [9]. Lyn is responsible for the phosphorylation of Tyr 359 and Syk is responsible for the phosphorylation of Tyr 8 and Tyr 21 [24–26]. Interestingly, all of those residues are located in the cytoplasmic domain of band 3.

Phosphatases (PTPs) have also been implicated in the phosphorylation of band 3 that follows oxidative stress [27–29] and inhibition of PTPs is due to the inhibitory Cys residue present in the catalytic site of some PTPs but the reactivity to H_2_O_2_ of the inhibitory Cys is 0.005-fold lower than GSH, indicating that, at its normal concentrations, GSH should very effectively protect PTPs from oxidative inhibition [30, 31]. Additional regulatory components could be also involved in the band 3 phosphorylation: Lyn kinase has been described to act as redox sensor [32]; Lyn activates Syk in different cell types and the role of Syk autophosphorylation remains to be elucidated [25, 26]. Moreover, all of those regulations have been mainly studied in immune cells and very little information is available on RBCs.

In the present report we performed a series of experiments to gain more information on the mechanisms that are involved in the Tyr phosphorylation of band 3 following a reversible membrane protein oxidation triggered by diamide and H_2_O_2_ and by hemichromes which cause irreversible oxidation.

## 2. Materials and Methods

### 2.1. Treatment of Red Blood Cells

Venous blood was drawn from healthy volunteers following informed consent and pelleted at 1000 g for 10 minutes at room temperature. After removal of the buffy coat, RBCs were again pelleted and washed 3 times with phosphate buffered saline (127 mM NaCl, 2.7 mM KCl, 8.1 mM Na_2_HPO_4_, 1.5 mM KH_2_PO_4_, 20 mM HEPES, 1 mM MgCl_2_, and pH 7.4) in 5 mM glucose (PBS glucose) to obtain packed cells. RBCs were suspended at 20% hematocrit in PBS glucose and incubated at 37°C in 0.5 mM diamide at different incubation times (0, 30, 60, 120, 240, and 360 minutes) and then in the presence of different diamide concentrations. Separate experiments were also performed in 5 mM H_2_O_2_ or 1 mM phenylhydrazine (PHZ). pH was measured after 180 minutes and adjusted to 7.4 with NaOH. When necessary to avoid tyrosine phosphorylation, RBCs were pretreated with 10 *μ*M of Syk inhibitor II (Calbiochem, USA), for 1 hour at 37°C in the dark, before oxidant treatment. For all protocols described, untreated controls were processed identically except that the inhibitor was omitted from the incubation. To prevent further phosphorylation of band 3, after incubation we washed the cells with cold buffer and membranes were immediately prepared.

### 2.2. RBC Membrane Preparation

Membrane proteins were prepared at 4°C on ice as previously described [9]. Briefly, 150 *μ*L of packed RBCs was diluted into 1.5 mL of cold hemolysis buffer (HB) (5 mM sodium phosphate, 1 mM EDTA, pH 8) containing a protease and a phosphatase inhibitor cocktail and then washed up to 4 more times in the same buffer (until membranes became white) in a refrigerated Eppendorf microfuge at 25000 g. The preparations were stored frozen at −80°C until use. Membrane protein content was quantified using the CD Protein Assay (Bio-Rad).

### 2.3. SDS-PAGE

To perform one-dimensional electrophoresis, membrane proteins were solubilized in Laemmli Buffer [33] in a volume ratio of 1 : 1. 10 *µ*g of proteins for analytical gels, 1 *µ*g of proteins for anti-band 3, and 30 *µ*g of proteins for anti-phosphotyrosine, anti-phosphoserine, and anti-Syk antibodies were loaded for western blot analysis and separated on 8% of polyacrylamide gel under reducing and nonreducing conditions. SDS-PAGE analysis was conducted by heating the sample for 5 minutes at 95°C and was run on the Bio-Rad mini-protean 3 setup.

### 2.4. Western Blot Analysis

Proteins separated by SDS-PAGE were transferred to nitrocellulose membranes as previously described [16] and then probed with 5 different antibodies: monoclonal anti-band 3 antibody (B9277, Sigma-Aldrich, Saint Louis, MO) produced in mouse (directed to cdbd3) diluted to 1 : 50000; anti-phosphotyrosine (sc7020, Santa Cruz, CA); anti-phosphoserine (ab9332, Abcam, Cambridge, UK); polyclonal anti-band 3 (sc20657); and anti-Syk (sc28337). The final two produced in rabbit from Santa Cruz, CA, all are diluted to 1 : 2000. Secondary antibodies conjugated to infrared fluorescent dyes excitable at 680 nm or 800 nm (IRDye: anti-mouse 800 CW 926-32210, anti-mouse 680 CW 926-32220, and anti-rabbit 800 CW 926-32211, Li-COR, USA) were then used to visualize the desired antigens with a laser scanner (Odyssey, Licor, USA). Quantitative densitometry study of tyrosine phosphorylation was carried out, and Syk translocation and band 3 oxidation levels were measured analyzing western blot images by the Odyssey V3.0 software, and values were expressed as arbitrary units.

### 2.5. Measurement of Band 3 SH-Groups Reactivity in the Presence of Increasing GSH Concentrations

RBC membranes were diluted in HB to obtain a 5 *µ*M band 3 concentration. Band 3 concentration was estimated measuring total membrane proteins in packed membranes (approximately 4 mg/mL) considering that band 3 represents approximately 25% of total membrane proteins and a band 3 M.W. of 95.000 Da. Resuspended membranes were incubated for 10 minutes on ice, with 0.1 mM diamide in the presence of increasing concentrations of GSH. The reaction was stopped by washing the solution 3 times with HB. The percentage of oxidized band 3 was evaluated by western blot following nonreducing 8% SDS-PAGE and expressed as percentage of the maximal oxidation measured in the absence of GSH. In the absence of GSH an average of 95.2 ± 4.5% of the band 3 was found present in reducible aggregates with a M.W. >200.000 KDa.

### 2.6. PTP Activity Measurement

Erythrocyte PTP activity was measured using phosphorylated band 3 as substrate. Phosphorylated band 3 was obtained treating RBCs with 1 mM diamide. Membranes were prepared and incubated for 10 min at 37°C with the cytoplasmic fraction of RBCs treated with different concentrations of diamide. 10 *μ*M Syk inhibitor II (Calbiochem, USA) was added to prevent further phosphorylation of band 3. The rate of band 3 dephosphorylation was expressed as PTP activity and as a percentage of maximal activity in untreated RBCs.

### 2.7. Hemoglobin Release Quantification

We used a simplified method to measure the relative changes of Hb in RBC cultures supernatant; after discarding RBC membranes by centrifugation, lysis was quantified by measuring hemoglobin absorbance at 405 nm in RBC supernatant and expressed in nmoles/mL [34].

### 2.8. Hemichrome Measurement

RBCs were solubilized in HB containing 1% Triton X-100, centrifuged at 15.000 g at 4°C. High molecular weight hemichrome aggregates were separated from the supernatant on a Sepharose CL-6B microcolumn. The hemichrome fraction was then diluted and quantified measuring heme absorbance at 560, 577, and 630 nm [35] and expressed as nmoles/mL of solubilized membranes.

### 2.9. Immunoprecipitation Studies

RBC membrane proteins were treated in the presence or the absence of 2 mM diamide, solubilized for 10 minutes on ice with 3 volumes of 1% Triton X-100 in HB. After centrifugation in a refrigerated Eppendorf microfuge at 15000 g, supernatants were collected and incubated with anti-mouse anti-band 3 cross-linked to Protein A-Sepharose (1 : 10) via bifunctional coupling reagent dimethyl pimelimidate for 2 hours at 4°C under gentle mixing. Beads were washed three times with 1% Triton X-100 in HB [9]. Laemmli buffer, containing 2% DTT (final concentrations), was added to packed beads (2 vol) and immunoprecipitated proteins were analyzed by immunoblotting using anti-band 3 and anti-Syk antibodies.

### 2.10. Cytoplasmic Domain of Band 3 Fragment Phosphorylation in Reconstituted Systems

To obtain the oxidized and nonoxidized cdbd3 fragment, RBCs were incubated with or without diamide (2 mM). Membranes were prepared as described above and cytoskeletal proteins were eliminated incubating the membranes with 0.1 M NaOH at 4°C (stripped membranes). Cdbd3 was then purified from RBC membranes as previously described [36]. The purity of cdbd3 was higher than 90%. After diamide treatment more than 60% of cdbd3 was present as disulfide cross-linked dimers. To measure band 3 phosphorylation in the presence of soluble oxidized and nonoxidized cdbd3, RBC membranes were incubated at 37°C for 10 minutes with RBC cytoplasm (diluted 1 : 10) as previously described [9]. The reaction was stopped by washing the membranes with HB. Band 3 tyrosine phosphorylation was then measured by western blotting as described above. The association between Syk and cdbd3 was tested incubating oxidized and nonoxidized cdbd3 with RBC cytoplasm at 37°C for 10 minutes and anti-band 3 immunoprecipitation was followed by western blot using anti-Syk antibody diluted 1 : 100.

### 2.11. Peptide Preparation for MS Analysis

Bands were excised from electrophoresis gels and were destained by doing several washes in 5 mM NH_4_HCO_3_/ACN (acetonitrile) (50/50 v/v) and successively dried with pure ACN. The gel slices were rehydrated for 45 minutes at 4°C in 20 *μ*L of 5 mM NH_4_HCO_3_ digestion buffer containing 10 ng/*μ*L of trypsin. Excess protease solution was removed and the volume was adjusted with 5 mM NH_4_HCO_3_ to cover the gel slices. Digestion was allowed to proceed overnight at 37°C.

### 2.12. Peptide Mass Fingerprinting by MALDI-TOF MS

Samples were loaded onto MALDI target using 1 *μ*L of the tryptic digests mixed 1 : 1 with a solution of CHCA (alpha-cyano-4-hydroxycinnamic acid) (10 mg/mL in ACN/TFA 0.1%, 40/60). MS analysis of peptides from 1-DE gel bands was performed with a MALDI-TOF micro MX (Micromass, Manchester, UK) according to the tuning procedures suggested by the manufacturer. Peak lists were generated with Proteinlynx Data Preparation using the following parameters: external calibration with lock mass using mass 2465.1989 Da of ACTH fragment 18-39 background was subtracted using the adaptive mode, performing deisotoping with a threshold of 3%. The MS spectra were converted into pkl files using Mass Lynx 4.0. Peak lists containing the 20 most intense peaks of the spectrum were sent to MASCOT PMF search (http://www.matrixscience.com/) using a Swiss-Prot database (release 50.0, 30 May 2006). Search settings allowed one missed cleavage with the trypsin enzyme to be selected, carboxymethylated cysteine as fixed modification and oxidation of methionine as potential variable modification and a peptide tolerance of 50 ppm. Only protein identifications with significant Mascot scores (*p* < 0.05) were taken in consideration.

## 3. Results 

### 3.1. Tyrosine and Serine Phosphorylative Response to Different Oxidant Species

Time dependent phosphorylative changes of the RBC membrane proteins have been measured comparing the effects of (i) diamide, a single electron oxidant that induces disulfide formation [9, 26], (ii) hydrogen peroxide (H_2_O_2_) that is physiologically generated from superoxide anion during methaemoglobin formation and by denatured hemoglobin products [37], and (iii) phenylhydrazine (PHZ) that reacts specifically with hemoglobin determining the formation of hemichromes which are capable of triggering ROS production [38, 39].

**Figure 1:**
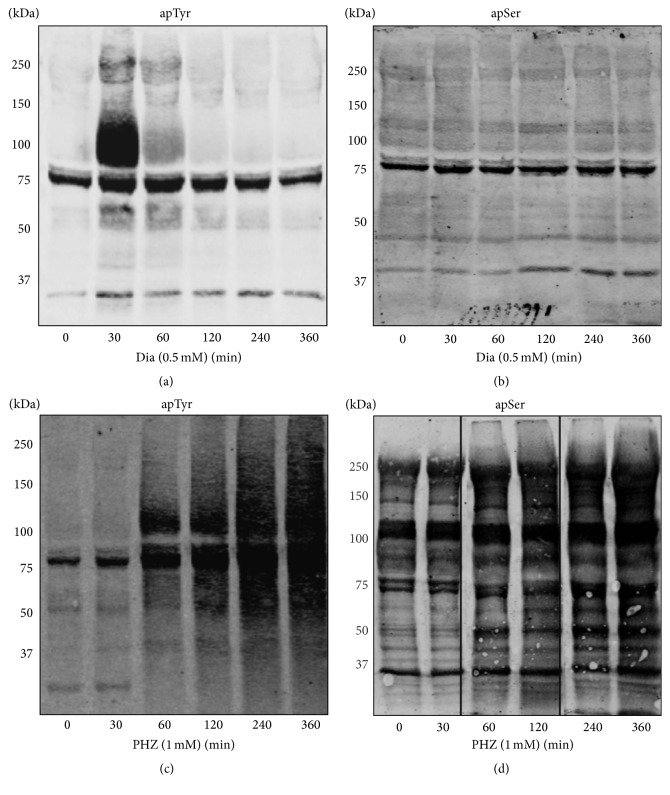
Time course of erythrocyte membrane proteins treated with oxidants. Erythrocytes were treated with 0.5 mM diamide (Dia) (panels (a) and (b)) and with 1 mM phenylhydrazine (PHZ) (panels (c) and (d)) at different incubation times. Membrane proteins were separated by 8% SDS-PAGE in the presence of reducing agent, blotted on nitrocellulose and stained with anti-phosphotyrosine (apTyr) and anti-phosphoserine (apSer) antibodies. Images were acquired using a laser IR fluorescence detector (Odyssey, Licor, USA). Results are representative of 4 separated experiments.

**Figure 2:**
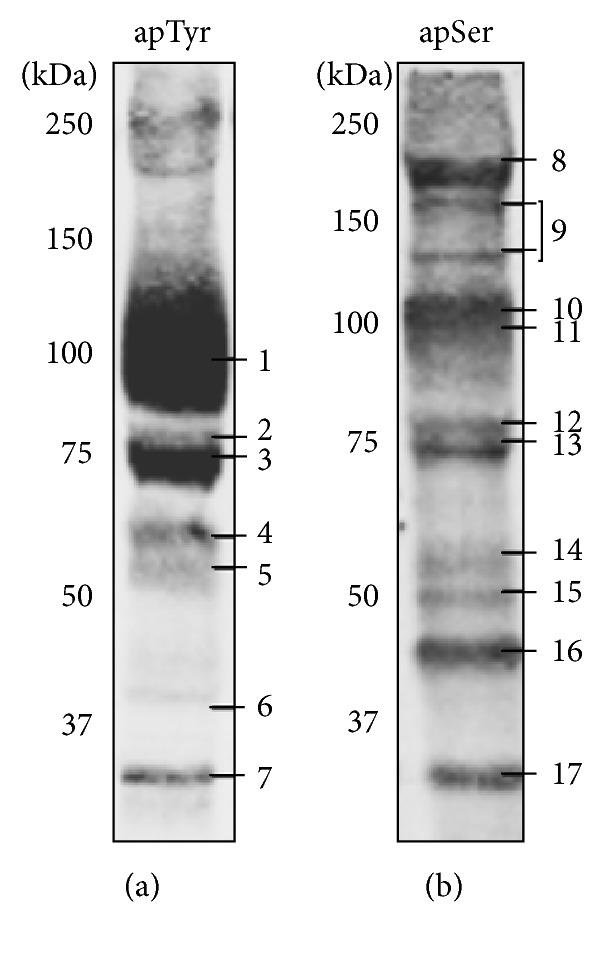
Protein phosphorylation analysis by mass spectrometry. Tyrosine phosphorylated proteins after 30-minute incubation of RBCs with 0.5 mM diamide (a) and serine phosphorylated proteins after 2-hour incubation of RBCs with 1 mM PHZ (b). Phosphorylated proteins were analyzed by mass spectrometry (MALDI-TOF) (Table 1). Band numbering in panels (a) and (b) identifies the proteins listed in Table 1.

Diamide caused an intense and transient Tyr phosphorylation of band 3 and of proteins 4.1 and 4.2 though to a lesser extent and Ser phosphorylation changes in additional membrane protein (Figures 1(a), 1(b), and 2 and Table 1). H_2_O_2_ induced a phosphorylation response identical to diamide but at 10-fold higher concentration (data not shown). This is plausibly due to the potent scavenging activity of catalase and glutathione peroxidase in RBCs on H_2_O_2_.

Conversely PHZ caused a slow phosphorylation response measurable only after 60 minutes of incubation (Figures 1(c) and 1(d)). Tyr phosphorylation of band 3 was one of the earliest events but additional proteins were also phosphorylated at Tyr and Ser residues after 120–360 minutes of incubation. These data were also supported by the absence of reactivity when the proteins were treated with *λ*-phosphatase, which was used to remove phosphate groups from blotted proteins (data not shown). Control experiments to exclude a direct oxidant effect of PHZ (2 mM) on isolated membranes revealed no effect on band 3 sulfhydryl groups (data not shown). The lack of short term effects of PHZ on membrane protein phosphorylation is coherent with its specific action on hemoglobin and the slow formation of hemichromes [38, 39]. A list of phosphorylated proteins, identified by mass spectrometry, is shown in Figure 2 and Table 1. At least one phosphorylation site has been identified on each phosphoprotein with the exception of protein 4.2. It should be noticed that similar protein phosphorylation patterns have been previously observed in pathological situations characterized by high content of hemicromes such as malaria infected RBCs, G6PD deficiency, and thalassemia [16, 18].

### 3.2. Functional Relationships between Band 3 Oxidation and Its Tyr Phosphorylation

**Figure 3:**
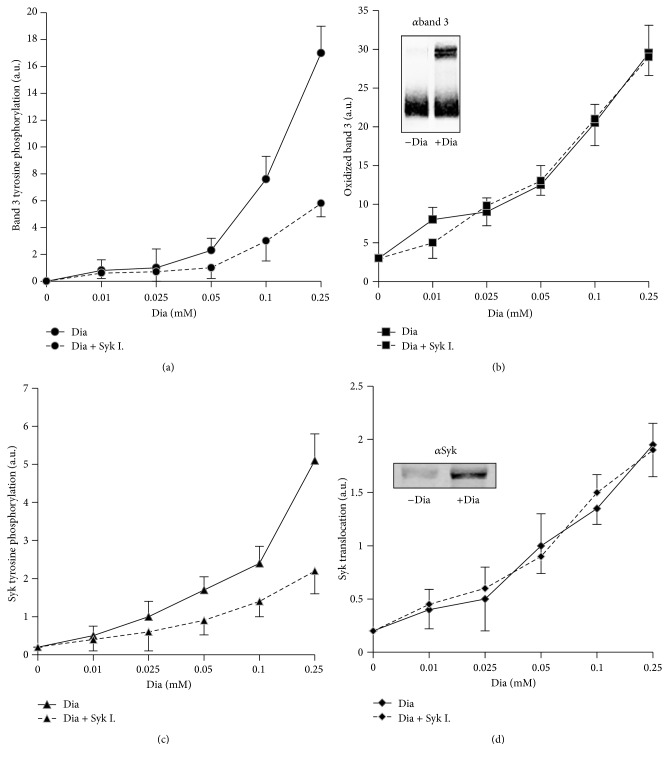
Band 3 modifications and Syk activation following diamide treatment. RBCs were treated with increasing concentration of diamide for 30 min in the presence or in the absence of 10 *µ*M Syk inhibitor II (Syk I.). Band 3 tyrosine phosphorylation (panel (a)). Band 3 oxidative crosslinking (oxidized band 3, Dia) expressed as the amount of oligomeric band 3 (apparent M.W. higher than 200 KDa) under nonreducing conditions (panel (b)). Syk tyrosine phosphorylation measured in whole cellular extracts (panel (c)). Syk bound to the membranes (Syk translocation) (panel (d)). Western blotting was quantified using an IR fluorescence detection scanner (Odyssey, Licor, USA). Images were analyzed by Odyssey V3.0 software. Values are representative of 4 separated experiments and are expressed as arbitrary units (au); the bars represent the standard deviations.

**Figure 4:**
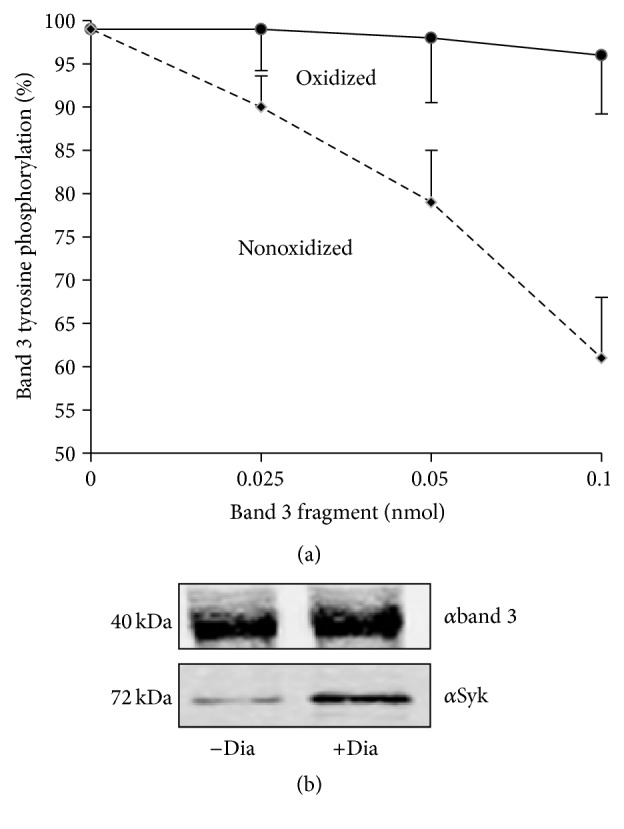
Competitive effect of oxidized and nonoxidized cdbd3 on band 3 phosphorylation and its association with Syk. Band 3 Tyr phosphorylation was measured in membranes obtained from diamide treated RBCs in the presence of RBC cytoplasm at increasing concentration of oxidized or nonoxidized cdbd3. The level of band 3 phosphorylation is displayed as percentage of its maximal phosphorylation absence of cdbd3 (panel (a)). Nonoxidized (−Dia) cdbd3 and oxidized (+Dia) cdbd3 were incubated with RBC cytoplasm and immunoprecipitated by anti-cdbd3 antibody. Immunoprecipitated proteins were western blotted with anti-band 3 (panel (a)) and anti-Syk antibody (panel (b)). Western blots are representative of 4 separated experiments.

To obtain quantitative data on the relationships intercurring between band 3 oxidation and its Tyr phosphorylation, we performed parallel measurements of the detection limits of band 3 phosphorylation (Figure 3(a)) and its oxidative crosslinking (Figure 3(b)) starting from very low concentrations of diamide. This experiment revealed that both events become measurable at the same diamide concentration (10–25 *μ*M) that, due to the buffering effect of cellular GSH, are not expected to exert an effect on protein thiols. We observed a dose-dependent increase of the phosphorylation signal (Figure 3(c)) and of the binding of Syk to the membrane (Figure 3(d)). Interestingly both phenomena became detectable in the same concentration range of diamide. Those data comprehensively indicate that, as previously suggested [9], Syk acts preferentially on oxidized band 3. To further investigate this hypothesis, we tested if purified oxidized cdbd3 fragment may exert a competitive inhibitory action on the phosphorylation of band 3.  Figure 4(a) shows that oxidized cdbd3 exerts a dose-dependent inhibitory effect on band 3 phosphorylation, while nonoxidized cdbd3 fragment did not exert any measurable effect. RBC membranes containing oxidized band 3 were used as PTPs substrate; on the contrary, experiment performed with purified band 3 provided unreproducible results possibly indicating that the requirement of a specific quaternary structure of oxidized band 3 is essential to allow the docking of Syk. Immunoprecipitation studies confirmed that Syk binds prevalently to the oxidized form of cdbd3 (5.8-fold higher, *p* < 0.01) while no significant difference has been observed between the amount of cdbd3 immunoprecipitated from oxidized and nonoxidized samples (Figure 4(b)).

### 3.3. Measurement of the Accessibility of the Band 3 Cys Residues

The characteristic accessibility of the two cysteins 201 and 317 located in the cytoplasmic domain of band 3 has been already demonstrated [36]. To obtain quantitative data on the relative accessibility of those Cys residues, we measured the effect of diamide (100 *μ*M) in forming band 3 (5 *μ*M) intermolecular disulfide bonds on increasing concentrations of GSH. This experiment showed that at 0.1 mM GSH concentration (20-fold higher than band 3) approximately 40% of band 3 was still oxidized by diamide; at 1 mM GSH concentration (200-fold higher than band 3) approximately 20% of band 3 was still oxidized (Figure 6(a)), indicating much higher accessibility of the Cys residues located in the cdbd3 than GSH. Those results are therefore in agreement with the observed oxidation of band 3 with low concentrations of diamide as the blood concentration of GSH is approximately 1 mM. Moreover, we have noticed that at low concentrations of diamide (50–100 *μ*M) no oxidation of GSH was detectable (data not shown).

### 3.4. Comparative Analysis of Syk Kinase and Protein Tyr Phosphatase Activities following Sulfhydryl Group Oxidation

Erythrocyte PTPs have been implicated in promoting the Tyr phosphorylation of band 3 due to an inhibitory Cys residue located in their catalytic domain [10, 40].

To rule out the possibility that diamide treatment, at concentrations that induce band 3 phosphorylation, may also determine a substantial inhibition of PTPs, we compared the levels of band 3 phosphorylation and PTP inhibition at different diamide concentrations.  Figure 6(b) shows that, after treating RBCs with 1 mM diamide that causes a nearly maximal band 3 phosphorylation, PTPs still display approximately 70% of their maximal activity. This finding is in agreement with the low reactivity of the regulatory Cys residue of PTPs that has been previously reported [30].

### 3.5. Effect of Syk Inhibitors on the Membrane Destabilization Induced by Phenylhydrazine 

**Figure 5:**
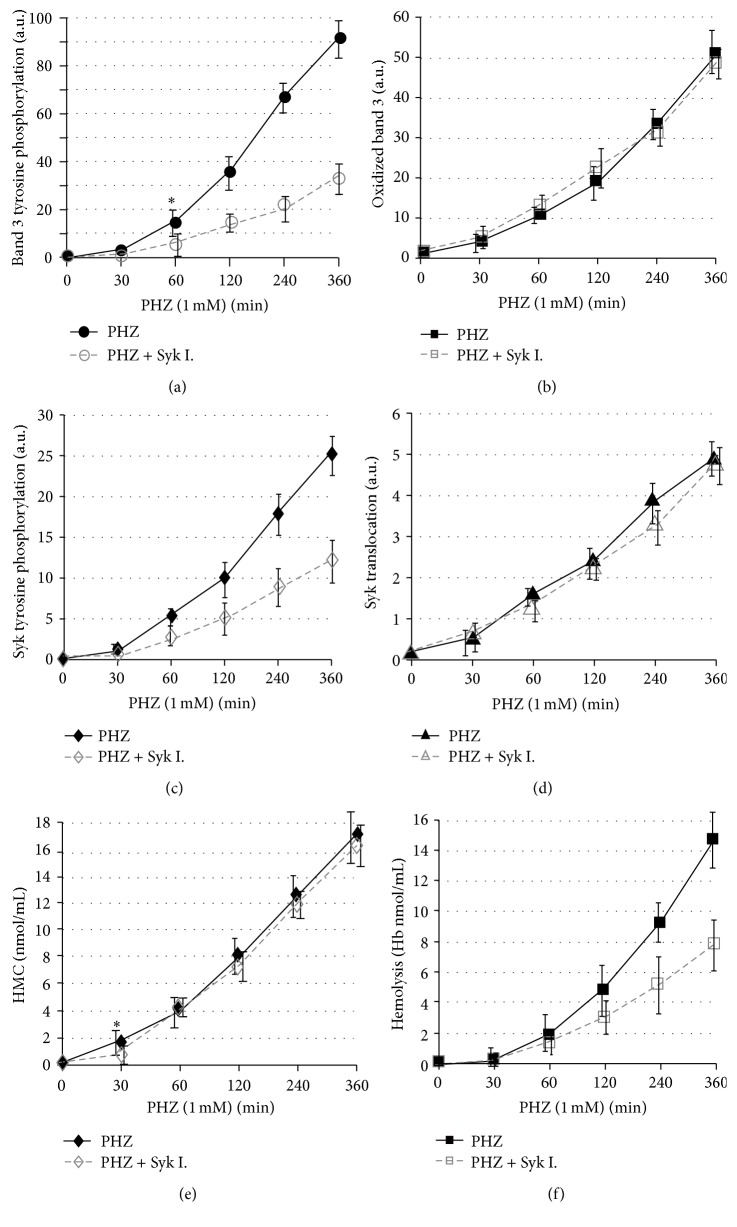
Band 3 modifications, hemichrome formation, and hemolysis after phenylhydrazine treatment. Erythrocytes were treated with 1 mM phenylhydrazine (PHZ) at different incubation times in the presence or in the absence of Syk inhibitor II (Syk I.). Band 3 tyrosine phosphorylation (panel (a)), oxidized band 3 (panel (b)), Syk phosphorylation (panel (c)), and Syk translocation (panel (d)) were quantified acquiring anti-phosphotyrosine, anti-band 3, and anti-Syk western blots using an IR fluorescence detection scanner (Odyssey, Licor, USA) and analyzing images with Odyssey V3.0 software. Values are the average of 5 separated experiments and are expressed as arbitrary units (au); the bars represent the standard deviations. Hemichromes (HMC) were quantified by vis spectrometry (panel (e)), hemolysisby measuring hemoglobin absorbance at 405 nm (panel (f)) and are expressed in nmoles/mL. *∗* indicates the minimal concentration or shorter incubation time that determines a statistically significant change by Student's *t*-test in comparison to the control sample (*p* < 0.01).

**Figure 6:**
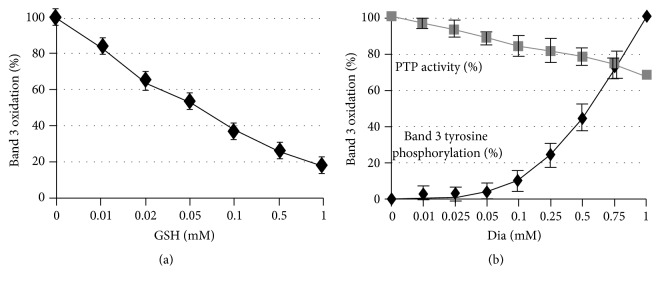
Comparative analysis of Syk kinase and protein Tyr phosphatase activities following diamide treatment. Erythrocyte membranes were treated with 0.1 mM diamide in the presence of increasing concentrations of GSH. Band 3 oxidation (percentage of maximal oxidation, in the absence of GSH) is showed in panel (a). Tyr kinase activity was measured as band 3 tyrosine phosphorylation at different diamide concentrations and expressed as percentage of maximal phosphorylation. PTP activity was measured as dephosphorylation of Tyr phosphorylated band 3 (obtained treating RBCs with 1 mM diamide) and expressed as percentage of maximal PTP activity (panel (b)). Band 3 oxidation and phosphorylation were quantified by Odyssey V3.0 software. Values are average of 3 separated experiments; the bars represent the standard deviations.

Differently from the effect of diamide that caused reversible changes, after phenylhydrazine treatment, band 3 oxidation, and its phosphorylation, Syk translocation to the membrane and its phosphorylation increased progressively, paralleling the generation of hemichromes (Figure 5). Also in this case Syk inhibitors markedly inhibited band 3 phosphorylation with no apparent effect on band 3 oxidation and Syk translocation (Figures 3 and 5).

Interestingly, Figure 5(f) shows that phenylhydrazine treatment causes a progressive leak of hemoglobin through the membrane. Hemolysis was substantially suppressed by Syk inhibitors indicating that irreversible band 3 phosphorylation may induce a progressive destabilization of the membrane plausibly through the weakening of the linkages between band 3 and cytoskeleton [23].

## 4. Discussion

RBCs rapidly react to oxidative stress through very intense Tyr phosphorylation of band 3, their major integral membrane protein. We previously found that the phosphorylation of band 3 affects its interactions with the cytoskeleton inducing membrane destabilization [23]. This phenomenon appears to play a central role in the release of membrane microparticles containing hemichromes from thalassemic erythrocytes and appears to be involved in the action mechanism of some prooxidant antimalarial compounds [18, 41]. Denatured hemoglobin products (hemichromes) are generated in senescent erythrocytes in numerous hemolytic diseases [42] and in malaria [16]. They bind to the cdbd3 and release iron in the membrane generating free radical species. Therefore, hemichromes appear to be a sensible source of redox stress under physiological conditions and in prooxidant pathological conditions. The crystal structure of cdbd3 indicates a tight dimer formed by interlocking dimerization arms of the two monomers. The Cys 201 residue in one subunit and the Cys 317 residue of the paired subunit are at close distance and can easily form intermolecular disulfide bonds following moderate oxidative stress [36, 43] and following the binding of hemichromes [18, 35]. No data are available on the structural changes induced by this modification but previous findings demonstrated an increased accessibility of extracellular band 3 epitopes following the exposure to very low concentration of oxidants and in senescent red cells [44–46], suggesting that conformational modification may occur following disulfide crosslinking of band 3.

Anyway, the involvement of Syk kinase of band 3 oxidation and of all the major steps of the pathway such as the mechanism of redox sensing, its transduction, the regulation of Syk activation, and docking to band 3 need to be clarified to envisage the physiological role of this intense redox response characteristic of erythrocytes. To address those issues, the present work has been performed to obtain a series of quantitative data to study (i) the temporal and dose effects of different physiological and nonphysiological oxidants in eliciting the minimal band 3 and Syk modifications, (ii) the role of disulfide cross-linked band 3 in docking Syk, (iii) the buffering effect of GSH on the oxidation of band 3 Cys residues to rule out if band 3 could display activity as redox sensor in intact erythrocytes, and (iv) the relative roles of Syk activation and docking versus PTPs inhibition in the phosphorylation of band 3.

The presented results indicate that band 3 possess highly reactive Cys residues capable of being easily oxidized in the presence of physiological concentration of GSH and that disulfide cross-linked band 3 docks Syk and acts as competitive inhibitor of band 3 phosphorylation. Those results support the observed changes in whole RBCs with very low concentration of a specific sulfhydryl reagent (diamide) or following the formation of minute amounts of hemichromes. In both models band 3 phosphorylation exactly parallels its oxidation. On the other hand erythrocytes seem to be fairly protected by H_2_O_2_. The comparative measurement of band 3 phosphorylation and of PTPs inhibition at different concentrations of diamide revealed that intense phosphorylation can occur at concentrations that minimally inhibit erythrocytes PTPs acting on phospho-band 3. This finding is in accordance with the relatively low reactivity of the Cys residue located in the catalytic site of PTPs [30] and with the much higher reactivity of the Cys residues located in the cdbd3.

In the present report, we observed that Syk inhibitors are potent inhibitors of the hemolysis that follows to the generation of hemichromes. Considering that treatment with diamide or H_2_O_2_ which induces a transient phosphorylation of band 3 does not cause hemolysis, a persistent phosphorylation of band 3 induced by irreversible hemichromes apparently leads to a severe membrane destabilization. It should be anyway noticed that hemichromes formation was also accompanied by serine phosphorylative changes involving some membrane protein; those phosphorylation changes have been usually considered to cause a decrease of the affinity between some components of the RBC membrane junctional complexes [47–50] and may therefore contribute to an alteration of the membrane structure.

Considering that band 3 phosphorylation may have a function in remodeling the RBC membrane to remove noxious hemichromes [18], the present findings support the hypothesis that erythrocytes may possess a very straight and effective mechanism to sense the oxidative stress exerted by low amounts of hemichromes through band 3 oxidation, selective docking of Syk, and phosphorylation of two band 3 Tyr residues critical for assuring the local stability of the membrane.

## Figures and Tables

**Table 1:** 

Band number	Protein name	Accession number	Phosphorylated peptide
1	Band 3	P02730	MEELQDD**Y**ED (Tyr 8) **Y**EDPDIPESQ (Tyr 21) PAKPDSSF**Y**K (Tyr 359)
2	Protein 4.1	P11171	V**Y**ECVVEKHA (Tyr 222)
3	Protein 4.2	P16452	Not identified
4	Catalase	P04040	KVWPHKD**Y**PL (Tyr 308)
5	P55	Q00013	AIRSQYAH**Y**F (Tyr 429)
6	Actin	P60709	GRDLTD**Y**LMK (Tyr 188)
7	G3PDH	P04406	PFIDLN**Y**MVY (Tyr 42)
8	Beta spectrin	P11277	ERT**S**PVSLW (Ser 2114)
9	Ankyrin	P16157	DQVVE**S**PAIP (Ser 856)
10	Alpha Adducin	P35611	REKSKKY**S**DV (Ser 408)
11	Beta Adducin	P35612	TP**S**FLKKSKK (Ser 713)
12	Protein 4.1	P11171	QEQYE**S**TIGF (Ser 461) RH**S**NLMLEDL (Ser 664)
13	Protein 4.2	P16452	LLNKRRG**S**VP (Ser 248)
14	Catalase	P04040	TFVQSG**S**HLA (Ser 517)
15	P55	Q00013	SC**S**PFGKKKK (Ser 243)
16	Actin	P60709	ANTVL**S**GGTT (Ser 300)
17	G3PDH	P04406	I**S**WYDNEFGY (Ser 312)

## Abbreviations

ROS: Reactive oxygen species
GSH: Glutathione
G6PD: Glucose 6 phosphate dehydrogenase
PTKs: Protein tyrosine kinases
PTPs: Protein tyrosine phosphatases
cdbd3: Cytoplasmic domain of band 3.
